# Editorial: Crop breeding involving epigenetic inheritance

**DOI:** 10.3389/fpls.2023.1239713

**Published:** 2023-07-20

**Authors:** Victor Raboy, Ryo Fujimoto

**Affiliations:** ^1^ Independent Researcher, Portland, OR, United States; ^2^ Graduate School of Agricultural Science, Kobe University, Kobe, Japan

**Keywords:** crop breeding, agronomic traits, epigenetics, yield stability, stress tolerance

It has become clear that epigenetic variation contributes to the inheritance of valuable traits in crop plants (**Figure 1**). This has led to many studies of the molecular mechanisms underlying epigenetic variation and its inheritance. However, there have been relatively few reports of translation of this new science to successful crop breeding. This Research Topic contains five papers, but only one of which is a research report describing the implementation of epigenetics as a tool in crop breeding (Ketumile et al.). Of the remainder, two are reviews (Tonosaki et al.; Gupta and Salgotra), and two are analyses that describe the contribution of epigenetics to a pre-existing trait or a hybrid (Chen et al.; Ma et al.). This reflects the current status of the literature in the field: there are a great number of reviews that extol the potential of epigenetics in crop breeding, and an even greater number of papers that describe the molecular biology behind previously known epigenetic traits, however there are very few research reports describing the novel implementation of epigenetics to crop breeding, whether it be successful or not.

**Figure 1:**
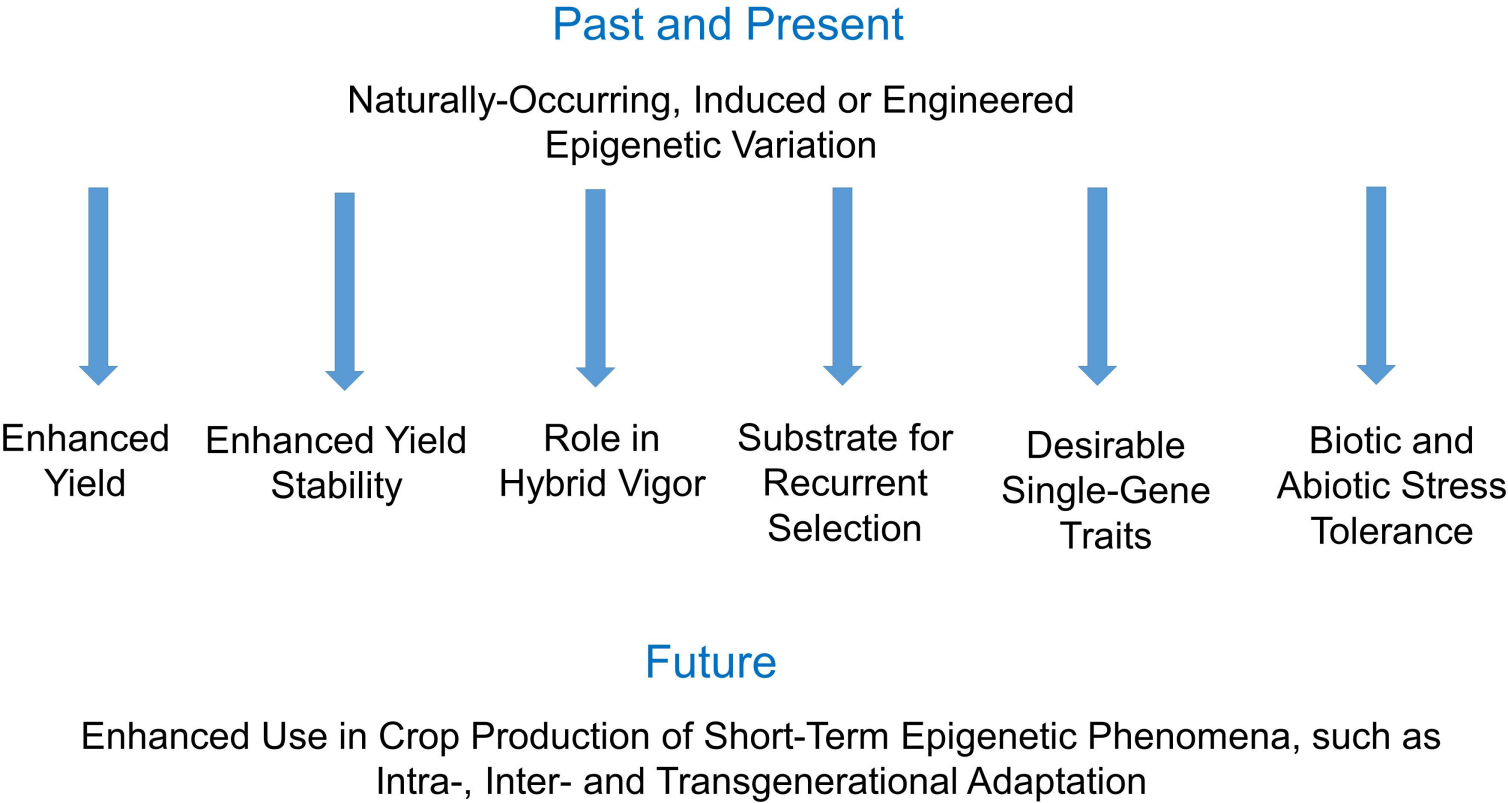
Crop breeding involving epigenetic inheritance: past, present and future.

Ketumile et al. describe recent progress in the application of a strategy that takes advantage of the positive impact on yield resulting from the increase in *de novo* epigenetic variation that in turn results from suppression of *MSH1* (*MutS Homolog 1*). Working with *Sorghum bicolor*, following RNAi-mediated suppression of *MSH1* and identification of RNAi-null progeny, the authors found that in the absence of any selection, the enhanced epigenetic variation resulted in a 10% yield boost under ideal field conditions and a 20% increase under “extreme low nitrogen” conditions. Furthermore, the authors report early-stage selection resulted in yield boosts of 36% under ideal field conditions and 64% under marginal field conditions. These exciting results clearly illustrate the potential for epigenetics in crop breeding and production. But perhaps more importantly, they illustrate that breeding that targets genome-wide epigenetic variation can sizably increase stability of crop yields across diverse production environments. This finding may in the end prove of great value for maintaining crop production during an era of climate change that results in increasingly challenging production environments.

Chen et al. elucidated the molecular mechanisms underlying enhanced Fe-deficiency response in the naturally-occurring tomato (*Solanum lycopersicum*) epimutant *Cnr* (*Colorless non-ripening*). This work demonstrated that in addition to other previously identified functions such as in fruit ripening, *SlSPL-CNR* (*Solanum lycopersicum Sauamosa promoter-binding protein-like*) is a novel regulator of Fe-deficiency response and identified Fe-deficiency responsive genes. Ma et al. added to the growing understanding of the role of epigenetics in the phenomenon of heterosis in crops, in particular with respect to allopolyploid crops. They found that in *Brassica napus*, differential parental transcriptome programming and histone modification when transmitted to offspring contributed to hybrid vigor.

Both Tonosaki et al. and Gupta and Salgotra provide thorough reviews of the molecular mechanisms underlying epigenetic traits in crops and provide lists of known cases of crop traits conditioned by epigenetic variation. These reviews contribute to the growing appreciation of the potential for enhanced crop breeding resulting from enhanced understanding of epigenetics. Beyond that, Tonosaki et al. also introduce an important distinction: that of the role of epigenetics in crop breeding *versus* crop production (**Figure 1**). Perhaps the role of epigenetics in crop breeding requires inheritance over multiple generations whereas a more short-term phenomenon like transgenerational adaptation to stress may play an important role in enhancing crop production across diverse environments.

In summary, it is likely that over the next decade or two the potential of the growing knowledge of epigenetics that underpins crop traits and productivity will be more fully realized, providing enhanced productivity and quality across diverse production environments. The growing understanding of the role of epigenetics in hybrid vigor will lead to updated strategies for hybrid seed production, such as updated approaches for selection of inbreds, or other approaches to generate beneficial novel epigenetic variation. Papers describing CRISPR-like targeting for epigenetic modification of selected target genes of agronomic or horticultural value will become more common. Similarly, enhanced appreciation for transgenerational adaptation in response to stress may lead to strategies to use this phenomenon in crop production. These advances could prove critically important to enhancing and stabilizing crop yields in this era of climate change and increasing scarcity of resources.

## Author contributions

All authors listed have made a substantial, direct, and intellectual contribution to the work and approved it for publication.

